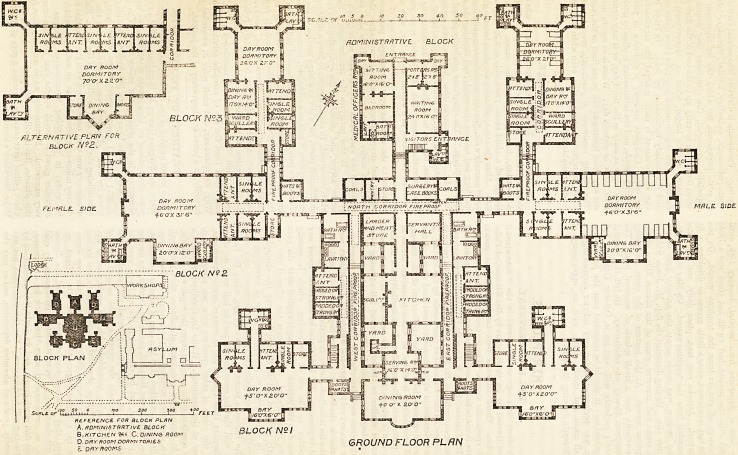# An Asylum Infirmary for 100 Beds

**Published:** 1889-11-16

**Authors:** Richard Greene

**Affiliations:** Medical Superintendent of the Berry Wood Asylum, Northampton


MODEL PLAN FOR AN ASYLUM INFIRMARY WITH ONE HUNDRED BEDS. By Dr. R. Greene.

### AN ASYLUM INFIRMARY FOR 100 BEDS.

By RICHARD GREENE, Medical Superintendent of the Berry Wood Asylum, Northampton.

"CHRONICLE your failures" is, or ought to be, a maxim both in medicine and in architecture, and as Dr. Howden has published in the January number of the *Journal of Mental Science* the successful design for the new hospital at the Montrose Asylum, I gladly avail myself of the invitation of the editor of THE HOSPITAL to hand him over one of the unsuccessful designs, thus giving everyone interested in asylum plans an opportunity of studying two of the first three in the competition asked for by the Montrose Board.

In the preparation of these plans I was associated with Mr. B. S. Jacobs, of Hull, the same architect who worked with me in designing the Derby Borough Asylum, lately opened. The plans were returned from Montrose without a single word, and Mr. Saxon Snell, the referee to whom the plans were sent by the authorities, could not, without a breach of the unwritten law, tell us the relative position in which these plans stood in his estimation; but the conditions issued to competitors stated that "the managers would avail themselves of an architect to advise with them in the selection of the best designs, and the author of the design placed first in order of merit shall be employed as architect to execute the new buildings on the usual terms of professional remuneration; and the author of the design placed second in order of merit shall be paid the sum of £25." As we did not execute the building, and as we did not get the £25, we may safely conclude that we formed Mr. Saxon Snell's "wooden spoon," as the plans were admittedly in the first three.

The conditions issued by the Montrose Board were chiefly: That the hospital was to contain 100 beds; that each division was to contain dayrooms, associated dormitories, single-bedded rooms, and a dayroom dormitory; that rooms for an assistant medical officer, for a matron, and for the usual number of attendants and servants were to be provided; that as far as possible the patients were to be accommodated on the ground floor; that a dining-room for sixty patients was desirable, and that the cost was to be about £10,000.

There was a difference of nine feet in the level of the ground between the north and south extremities of the building, and this difference was met by a series of steps arranged so as to interfere as little as possible with the general communications. No. 3 Blocks and the administration block are on the high level. No. 2 Blocks and their connecting corridors are on the intermediate level, and No. 1 Blocks, with the dining-hall and kitchen, are on the low level.

The various blocks may appear somewhat near to each other on plan; but the nature and size of the site does not admit of a more extended grouping without obstructing the view from a portion of the existing main building, besides which, when it is taken into consideration that the buildings generally are only of one story high, it will be found that there is ample air space and light for each block, and that the dayrooms have each an unobstructed view.

The accompanying plan consists of eight blocks, each block being complete in itself, and separated from the others by fireproof corridors.

The centre block to the south contains the dining-hall, having separate entrances for men and women, and having attached to it the serving-room, the kitchen, scullery, servants' hall, and all other offices required for the domestic department. To the north of this is the administration block proper. It contains, on the ground floor, a sitting-room, bedroom, bathroom, etc., for the resident medical officer; a small sitting-room and bedroom for the hall porter, a large visiting-room, a surgery and case-book room, pantry and storeroom for the staff, and coal store. On the first floor are the sitting-room, bedroom, and bathroom for the matron, rooms for the cook, domestic servants, housemaids' closet, etc.

To the east of the dining-hall block is a block for twenty-two patients. On the ground floor are the dayrooms, three single rooms, attendants' room, and storerooms. It often happens that one or two patients are so noisy that they ought not to be placed near the others at night, and for these cases there are two single rooms adjoining this ward, but approached by a separate corridor. Close to these are an attendant's room, which might be allotted to the night attendant, and a bathroom and dressing-room. The latter have a door of communication, and are so placed that they can be easily reached by patients from both blocks No. 1 and No. 2. The block under description, which is No. 1 Block, is the only part of the hospital occupied by patients which has a first floor, and here it is intended for sleeping room only. There is ample space for fourteen patients in the dormitory, and the three single-bedded rooms adjoining, and the five single-bedded rooms on the ground floor, provides the necessary space for twenty-two patients. It will be seen that this block has perfect cross ventilation from end to end, and a very simple arrangement in the construction of the single rooms ensures efficient cross ventilation in the width also. This system has been carried out in several large dormitories in the Berry Wood Asylum with the result that these rooms are practically as good as pavilion wards, and this was an important consideration for the Montrose Hospital, as it was understood to be intended for the treatment of the physically sick.

To economise heat as far as possible, the fire-places are arranged in the internal walls.

The closets, slop-sinks, and small room for pails, etc., are placed in a small block by themselves, connected to the ward by a short corridor, having cross ventilation. It may be confidently stated that no bad smell can enter the wards; and the principle of having the w.c.'s projecting from the dayrooms and dormitories is followed in all the other blocks. In fact, the advantages of this system and the dangers of the old one have been so often demonstrated that it is unnecessary to do more than call attention to it.

The next or No. 2 Block is a dayroom dormitory, and there is sufficient space for eighteen patients. Fourteen sleep in the large room, and there are four single rooms, two attendants' rooms, ward scullery, store-room, bathroom, lavatory, and w.c.'s, etc. The block is designed in accordance with the arrangements now often recommended. As, however, difference of opinion exists on this point an alternative plan was shown, and one which I am convinced is not only more cheerful and comfortable, but also more convenient to manage, being more easily supervised. This alternative plan was of the same size, and accommodated the same number of patients. Twelve were to sleep in the large room, and there were six single rooms which opened into the dayroom dormitory.

The patients using these dayroom dormitories are for the most part too weak and ill to be taken to the general bathroom, and, therefore, a small bathroom is attached to the block. The w.c.'s occupy a small block at the opposite corner, and, as already remarked when speaking of No. 1 Block, are cut off from the ward by a short ventilating passage.

The next, or No. 3 Block, has been designed in the belief that it is desirable that every asylum should have a ward which, in case of emergency, could be entirely separated from the rest of the building, and treated as a distinct hospital. This is therefore arranged for ten beds, eight in the dayroom dormitory, and two in single rooms. There is a dining (or day) room and a small kitchen adjoining it, also two attendants' rooms and a storeroom.

The w.c.'s and bathrooms are on the same principle as those in No. 2 Block. It will be noticed that the bathroom contains a tank for rinsing and boiling, so that in the event of infectious disease the clothing could be at once disinfected and boiled. The block is provided with a separate entrance, and by master-locking the corridor door it could be completely severed from the main building. Each block is provided with a room for hats and boots.

The blocks on the west need not be described as they are counterparts of those on the east.

Should subsidiary water-tanks be considered necessary, it was proposed to place them in the towers which are built over the staircases in No. 1 Blocks.

The accommodation, exclusive of attendants' rooms, is as follows :

| | | |
|---|---|---|
| No. 1 block on male side.................... | 22 | beds. |
| No. 1 block on female side.............. | 22 | ,, |
| No. 2 block on male side.................... | 18 | ,, |
| No. 2 block on female side.............. | 18 | ,, |
| No. 3 block on male side.................... | 10 | ,, |
| No. 3 block on female side.............. | 10 | ,, |
| Total .................................... | 100 | ,, |

Mr. Jacobs carefully estimated the cost of the buildings, and was satisfied they could be well and substantially built for £10,000. That is at the rate of £100 a bed. In confirmation of this I may state that the total cost of the Derby Borough Asylum was £105 per bed, and this included a large recreation-room, chapel, superintendent's house, exceptionally large administration block, workshops, laundry, engine and boilers, heating apparatus, etc. Unlike all other asylums, the Derby Borough one was built for a sum considerably less than the estimate. In fact, the estimate of £10,000 is in all probability a high one. Although designed for an asylum hospital, it is manifest that very slight modifications would make it suitable for a general infirmary.

---

The percentage of alcohol in liqueurs has recently been proved to be very high, though it varies considerably. Carmelite has as much as 93 per cent. of alcohol in its composition, whereas Maraschino has only 30 per cent., Swiss absinthe has 70 per cent., green Chartreuse 62, white Chartreuse 43, Liqueur Rum 53, Kummel 40, Benedictine, Danzig, Goldwasser, and Curaçoa contain almost an identical amount—viz., 34, 32, and 32 respectively, but the percentage of alcohol in Curaçoa is found to vary from 32 to 51.

## Figures and Tables

**Figure f1:**